# Changes in sensory quality characteristics of coffee during storage

**DOI:** 10.1002/fsn3.35

**Published:** 2013-05-04

**Authors:** Michaela T L Kreuml, Dorota Majchrzak, Bettina Ploederl, Juergen Koenig

**Affiliations:** Department of Nutritional Sciences, University of ViennaAlthanstraße 14, Vienna, 1090, Austria

**Keywords:** Coffee, quantitative descriptive analysis, sensory changes, storage

## Abstract

How long can roasted coffee beans be stored, without reducing the typical coffee flavor which is mainly responsible for consumers’ enjoyment? In Austria, most coffee packages have a best-before date between 12 and 24 months, but it is not regulated by law. Therefore, there is the need to evaluate changes in sensory qualities of coffee beverages prepared from stored coffee beans. For preparation of the coffee beverages, the paper filter method was used. In the quantitative descriptive analysis (QDA) 10 trained assessors evaluated the intensity of 30 coffee attributes after roasting at the 9th and 18th month of storage, respectively. The sensory evaluation results showed reduction in the sensory qualities of coffee beverages after 9 months storage of roasted coffee beans. The positive associated odor and flavor attributes decreased in their intensity, whereas the negative associated odor and flavor attributes increased significantly (*P* < 0.05). After 18 months of storage, the rancid odor and flavor which indicate oxidation processes were even considerably perceivable. Consequently, we can assume that changes in sensory quality characteristics of roasted and vacuum-packed coffee beans during storage are possible.

## Introduction

Coffee is one of the most widely consumed beverages throughout the world for its unique sensory properties and physiological effects. Studies have shown that there is a great interest on shelf life of roasted and ground coffee (Ross et al. 2006; Manzocco and Lagazio 2009; Makri et al. 2011; Borém et al. 2013; Toci et al. 2013). Roasting is responsible for the flavor development and is a time-temperature-dependent highly complex process, whereby hundreds of chemical reactions and changes occur simultaneously. These changes include the Maillard and Strecker reactions, degradation of polysaccharides, proteins, trigonelline, and chlorogenic acids. The chlorogenic acids (CGA), a phenolic compound, are known to be responsible for astringency, coffee pigmentation, and aroma formation. Furthermore, thermal degradation during the roasting process of CGA also contributes to bitterness (Ky et al. 2001; Farah et al. 2006). Caffeine, a xanthine derivative may also cause a characteristic bitter taste depending on concentration (Casal et al. 2000; Ky et al. 2001; Farah et al. 2006; Keast 2008; Toci et al. 2013). In general, Robusta coffee is known for a bitter taste and astringency, while Arabica develops a fine acidity, better flavor, and is more intense in overall aroma (Ky et al. 2001; Nebesny and Budryn 2006). Not only roasting process induces transformation of chemical contents also during storage, roasted beans are susceptible to further chemical and physical changes that may greatly affect the sensory quality of coffee beverages. Water (moisture), air (oxygen), light, and extraneous odors have the greatest influence on quality of coffee during storage. Massive odor- and flavor losses are the consequence of the water solubility of essential oils of coffee and the formation of volatile flavoring substances with oxygen (Ross et al. 2006; Makri et al. 2011; Toci et al. 2013).

There are only few studies and data available on sensory profiling of coffees during storage (Ross et al. 2006; Manzocco and Lagazio 2009; Makri et al. 2011; Borém et al. 2013; Toci et al. 2013). The authors investigated the sensory changes of coffee beverages made out of roasted and ground coffee during the storage time in different periods, but not longer than 12 months. In Austria, most coffee packages have a best-before date between 12 and 24 months, but this is not explicitly regulated by law. Therefore, there is the need to investigate how long roasted coffee beans can be stored without reducing the typical flavor of coffee beverages, which is mainly responsible for the consumers’ enjoyment.

## Material and Methods

### Coffee samples

Two green coffee samples, 20 kg Arabica from Ethiopia growing area Limu and 10 kg Robusta from Vietnam, provided from the Austrian Coffee Institute (Hofwiesengasse 48, 1130 Vienna), were evaluated in this study. The samples were roasted in a shop roaster with a volumetric capacity of 12 kg at 163–165°C, 10 min for Arabica and 13 min for Robusta, respectively, resulting in a medium roasting degree (golden-brown color). Temperature variation inside the roaster was monitored using a thermometer during the roasting procedure. Due to the large quantity, Arabica coffee was roasted in two loads (Table 1).

**Table 1:** Roasting conditions of evaluated coffee species

	Arabica Ethiopia first load	Arabica Ethiopia second load	Robusta Vietnam
Roasting date	25 March 2009	25 March 2009	25 March 2009
Roasting temperature (°C)	Initial Temp.: 79 Max. Temp.: 165	Initial Temp.: 80 Max. Temp.: 163	Initial Temp.: 80 Max. Temp.: 164
Roasting time (min)	10.40	10.54	13.16
Degree of roast	Golden brown	Golden brown	Golden brown
Weight green coffee (kg)	10.50	10.54	10.32
Weight after roasting (kg)	8.97	9.17	8.56

Roasted coffee beans were packed under vacuum conditions in commercially available packages and stored in the dark at ambient temperature over a period of 18 months. Freshly roasted, 9 and 18 months stored coffee beans were ground with a manual coffee grinder (Zassenhaus, Solingen, Germany) and brewed. For preparation of coffee beverages the paper filter method was used (Melitta orginal 1 × 4). The coffee brews were percolated from 50 g of each ground-roasted coffee variety with a volume of 500 mL water using a filter coffeemaker (Philips coffee maker HD 7563/20 1000 W).

### Sensory evaluation

Sensory evaluation of the investigated coffee beverages was performed by quantitative descriptive analysis (QDA) according to Stone et al. (1974). It was conducted by a trained panel of 10 assessors (ISO-Standard 8586 2012) aged 20–35 years with ample experience in sensory evaluation of coffee, recruited from students of the Department of Nutritional Sciences, University of Vienna. The training consisted of a theoretical education, exercises in general sensory evaluation, and description of sensory attributes of coffee. Additionally, an individual questionnaire composed of questions about the eating habits and availability for the study was conducted. The overall coffee odor and flavor as well as the attributes brew-like, roasty, fruity/aromatic, and the sweet taste were defined by the consumers as positively associated descriptors, which characterize a good coffee quality. Woody, earthy, hay like in odor, and flavor as well as bitter taste and aftertaste and astringency were defined as negatively associated descriptors. The analysis took place at three times and occurred immediately after roasting, and after 9 and 18 months of storage, in individual booths at the sensory laboratory of University of Vienna, designed and equipped consistent with ISO-Standard 8589 (2007). The term list generated from trained assessors included five primary sensory categories such as appearance, odor (orthonasal perception), flavor and taste, mouthfeel, and aftertaste. Every category was described by certain descriptors and the vocabulary list contains 30 attributes (Table 2). The selection and definition of the descriptors were compiled in accordance with the papers by Han-Seok et al. (2008), Andueza et al. (2003), Narain et al. 2003, and ICO – International Coffee Organization (2002). During the quantitative assessment, the assessors evaluated the intensity of the specified attributes on a numeral ordinal 10-unit scale with the end values labeled as imperceptible and very intense. The coffee samples were served in a randomized order and evaluated by QDA three times over two sessions, in the morning and in the afternoon, respectively.

**Table 2:** Brewed coffee sensory attributes and their definitions

Categories	Descriptors	Definitions
Appearance	Color	The intensity of color
	Opacity (cloudiness)	Nontransparent or cloudy status
	Oiliness	Greasy and coating appearance
Odor (orthonasal perception)/flavor	Overall coffee odor/flavor	Aromatics associated with common coffee aroma
	Brew-like	Aromatics associated with freshly brewed roasted coffee
	Roasty	Aromatics associated with freshly roasted coffee beans
	Fruity/aromatic	Lightly sour and sweet aromatics associated with several fruits
	Burnt/smoky	Aromatics associated with burnt rice or something scorched or burnt
	Woody	Aromatics associated with wooden materials
	Earthy	Aromatics associated with soil or clay freshly cut grass or hay during biting
	Hay-like (grassy)	Aromatics associated with freshly cut grass or hay during biting
	Staleness	Aromatics associated with moldy, old coffee
	Rancid	Aromatics associated with oxidations processes of fat
Taste	Sweet	A basic taste associated with sucrose solution
	Sour	A basic taste associated with citric acid solution
	Bitter	A basic taste associated with caffeine solutions
Mouthfeel	Viscosity	Sticky characteristics to palate or mucosal surface in the oral cavity
	Astringent	Feelings associated with dry sensation associated with immature permissions or black/green tea
Aftertaste	Overall coffee	Long-lasting overall impression 1 min after having swallowed the coffee beverage
	Bitter	Long-lasting bitter taste 1 min after having swallowed the coffee beverage

### Statistical analysis

For each tested coffee species at each examination date, the mean value and standard deviation for each investigated attribute was calculated from the data of 10 panelists and two repetitions, altogether 20 individual measurements. For all calculations MS Excel 2003 (Microsoft, Redmond, WA) was used.

For determination of the statistical significance of differences between the analyzed coffees, the statistical software SPSS 15.0 (Statistical Package for the Social Sciences, SPSS Inc., Chicago IL) was applied. Primarily, the K-S-Test (Kolomogorov-Smirnov-Test) was used to show if the data were normally distributed (*P* > 0.05) or not (*P* < 0.05). Subsequently, normal distributed data were analyzed with t-test for independent samples or ANOVA (analysis of variance), and for not normally distributed data the Mann–Whitney test, was applied. Differences were regarded as statistically significant at *P* < 0.05.

## Results

### Quantitative descriptive analysis (QDA)

The results of QDA demonstrated large significant differences in the intensity of all evaluated sensory attributes in both investigated coffee cultivars after storage (Table 3).

**Table 3:** Sensory evaluation (QDA)^1^ results of brewed coffee prepared with fresh and stored coffee beans (mean ± SD).^2^

	Arabica^2^			Robusta^2^		
		
		Stored			Stored	
				
Attributes	Freshly roasted	9 months	18 months	Freshly roasted	9 months	18 months
Odor						
Overall coffee	8.13^Aa^ ± 0.80	7.10^Bb^ ± 1.34	5.50^Cb^ ± 0.76	7.47^Ac^ ± 1.48	6.22^Bd^ ± 2.01	4.60^De^ ± 1.73
Brew-like	7.78^Aa^ ± 1.24	6.50^Bb^ ± 1.09	4.25^Cc^ ± 0.99	5.17^Dd^ ± 1.82	2.82^Ee^ ± 1.47	2.10^Fe^ ± 0.64
Roasty	7.02^Aa^ ± 1.30	6.06^Bb^ ± 1.28	4.70^Cc^ ± 1.77	5.29^Dd^ ± 1.77	3.29^Ee^ ± 1.56	2.45^Fe^ ± 1.15
Fruity/aromatic	7.27^Aa^ ± 0.99	6.50^Bb^ ± 1.15	4.30^Cc^ ± 2.00	1.73^Dd^ ± 1.43	1.29^Ed^ ± 1.36	1.10^Fd^ ± 0.64
Burnt/smoky	1.48^Aa^ ± 1.36	2.00^Ba^ ± 1.68	4.50^Cb^ ± 1.39	6.53^Dc^ ± 1.45	7.22^Ec^ ± 1.29	8.20^Fd^ ± 0.77
Woody	0.78^Aa^ ± 0.75	2.16^Bb^ ± 1.22	3.20^Cc^ ± 1.01	5.40^Dd^ ± 1.10	7.15^Ee^ ± 1.63	7.73^Fe^ ± 0.97
Earthy	0.54^Aa^ ± 0.56	1.73^Bb^ ± 1.08	2.85^Cc^ ± 1.09	5.13^Dd^ ± 1.15	7.24^Ee^ ± 1.48	7.63^Fe^ ± 0.96
Hay-like	1.16^Aa^ ± 1.16	2.30^Bb^ ± 1.46	3.40^Cc^ ± 0.82	5.01^Dd^ ± 1.08	5.22^Ed^ ± 0.91	6.55^Fe^ ± 1.00
Staleness	0.00^Aa^ ± 0.00	1.81^Ab^ ± 1.29	4.18^Ac^ ± 2.34	0.13^Ad^ ± 0.26	6.46^Ae^ ± 1.90	7.35^Ae^ ± 0.73
Rancid	0.00^Aa^ ± 0.00	1.03^Bb^ ± 1.16	3.70^Cc^ ± 0.57	0.00^Dd^ ± 0.00	4.26^Ee^ ± 2.66	6.35^Fe^ ± 0.67
Flavor						
Overall coffee	8.01^Aa^ ± 1.00	7.17^Ab^ ± 1.11	6.40^Ac^ ± 0.94	6.78^Ad^ ± 2.08	5.81^Ade^ ± 2.37	4.00^Ae^ ± 1.86
Brew-like	7.34^Aa^ ± 1.32	6.19^Bb^ ± 1.61	4.70^Cc^ ± 1.49	4.15^Dd^ ± 2.26	2.87^Ee^ ± 1.71	2.30^Fe^ ± 1.72
Roasty	7.11^Aa^ ± 1.41	6.02^Bb^ ± 1.76	4.90^Cc^ ± 1.74	4.52^Dd^ ± 2.33	2.91^Ee^ ± 1.76	2.25^Fe^ ± 1.33
Fruity/aromatic	6.90^Aa^ ± 1.11	6.40^Ba^ ± 1.77	3.85^Cb^ ± 1.53	0.94^Dc^ ± 1.08	0.79^Ecd^ ± 0.85	0.30^Fd^ ± 0.44
Burnt/smoky	1.43^Aa^ ± 0.86	2.36^Ba^ ± 1.94	4.30^Cb^ ± 1.22	6.27^Dc^ ± 1.62	7.11^Ecd^ ± 2.77	7.75^Fd^ ± 1.07
Woody	1.05^Aa^ ± 0.97	2.02^Bb^ ± 1.16	3.55^Cc^ ± 1.05	5.81^Dd^ ± 1.12	7.24^Ee^ ± 1.58	8.15^Fe^ ± 0.59
Earthy	0.71^Aa^ ± 0.74	1.84^Bb^ ± 1.14	3.70^Cc^ ± 0.91	5.18^Dd^ ± 1.24	7.40^Ee^ ± 1.62	8.15^Fe^ ± 0.80
Hay-like	1.05^Aa^ ± 0.85	2.52^Bb^ ± 1.51	4.15^Cc^ ± 1.09	4.72^Dd^ ± 1.19	5.03^Ed^ ± 1.12	6.65^Fe^ ± 1.23
Staleness	0.00^Aa^ ± 0.00	1.88^Bb^ ± 1.56	4.25^Cc^ ± 1.80	0.07^Ad^ ± 0.15	6.91^De^ ± 1.94	7.30^Fe^ ± 0.98
Rancid	0.00^Aa^ ± 0.00	0.92^Bb^ ± 1.27	1.90^Cc^ ± 1.17	0.00^Ad^ ± 0.00	3.47^De^ ± 2.91	4.55^Fe^ ± 0.94
Taste						
Sweet	2.95^Aa^ ± 1.27	2.75^Ba^ ± 1.29	1.70^Cb^ ± 1.02	0.54^Dc^ ± 0.74	0.48^Ec^ ± 0.71	0.15^Fc^ ± 0.37
Sour	3.04^Aa^ ± 1.60	3.92^Ba^ ± 2.31	5.00^Cb^ ± 0.78	2.11^Ac^ ± 1.94	2.37^Dc^ ± 1.94	4.35^Cd^ ± 1.66
Bitter	3.55^Aa^ ± 1.62	4.14^Ba^ ± 0.51	6.10^Cb^ ± 1.11	7.36^Dc^ ± 0.95	8.39^Ed^ ± 1.33	8.75^Fd^ ± 0.47
Appearance						
Color	6.53^Aa^ ± 1.78	7.72^Bb^ ± 1.12	8.45^Cb^ ± 0.90	7.71^Dc^ ± 1.97	8.08^Bc^ ± 1.33	9.03^Ed^ ± 0,62
Opacity	3.46^Aa^ ± 2.39	5.26^Ab^ ± 2.20	6.73^Ab^ ± 0.91	4.81^Ac^ ± 2.51	5.76^Acd^ ± 2.50	6.43^Ad^ ± 0.99
Oiliness	1.93^Aa^ ± 1.91	4.07^Ab^ ± 2.41	4.98^Ab^ ± 1.81	1.77^Ac^ ± 1.95	3.34^Ad^ ± 1.92	4.08^Ad^ ± 1.17
Mouthfeel						
Viscosity	3.30^Aa^ ± 0.80	3.79^Ba^ ± 1.50	4.75^Cb^ ± 0.77	3.21^Ac^ ± 1.45	3.38^Bc^ ± 1.54	3.60^Dc^ ± 1.29
Astringent	3.37^Aa^ ± 2.06	4.11^Ba^ ± 1.10	5.30^Cb^ ± 1.38	5.83^Dc^ ± 1.75	6.22^Ec^ ± 1.46	7.38^Fd^ ± 1.06
Aftertaste						
Overall coffee	6.85^Aa^ ± 1.82	6.18^Bab^ ± 1.75	5.55^Cb^ ± 1.04	6.66^Ac^ ± 1.83	6.32^Bc^ ± 1.95	4.40^Dd^ ± 1.57
Bitter	3.24^Aa^ ± 1.10	3.68^Ba^ ± 0.63	5.40^Cb^ ± 1.14	6.19^Dc^ ± 1.02	7.38^Ed^ ± 1.29	8.05^Fd^ ± 0.83

1 QDA, quantitative descriptive analysis: The evaluation of the intensity of the descriptors was done using a 10-unit scale (0 = imperceptible and 10 = very intense).

2 Values represent the mean of 20 results ± SD, standard derivation (10 panelists, 2 repetitions).

Capital letters compare Arabica and Robusta treatments, and lowercase letters compare treatments within the variety. Same letters in the same row mean values are not significantly different, whereas different letters in the same row mean values are significantly different.

### Impact of storage on the sensory attributes of evaluated coffee species

The coffee brews prepared from freshly roasted Arabica and Robusta beans showed significant (*P* < 0.05) higher intensities in positively associated odor and flavor attributes, such as overall coffee odor and flavor, brew-like, roasty, and fruity/aromatic as well as the sweet taste than samples brewed from coffee beans stored for 9 and 18 months. Coffee beverages prepared from the 18 months stored beans indicated the lowest intensities of positive coffee descriptors. In contrast, the negative associated odor and flavor attributes like burnt/smoky, woody, earthy, and hay like as well as the astringency, bitter taste, and aftertaste increased significantly (*P* < 0.05) during storage. In addition, staleness and rancid odor and flavor, which indicate oxidation processes, were particularly perceivable after 18 months of storage (Table 3).

### Differences in sensory changes between Arabica and Robusta during storage

Both coffee varieties, Arabica and Robusta showed significant differences in the intensity of all evaluated sensory descriptors at the beginning of investigation and during storage. Coffee beverages prepared from Arabica beans demonstrated a significant (*P* < 0.05) higher intensity of positive attributes compared to Robusta coffee at each point of assessment. After 18 months of storage, these positively associated descriptors decreased significantly (*P* < 0.05) in both coffee cultivars, however, more evident in Robusta than in Arabica. The negatively associated odor and flavor attributes like burnt, woody, earthy, and hay like, as well as astringency, the bitter taste, and aftertaste were significantly higher (*P* < 0.05) in the Robusta samples than in the Arabica, even in coffees prepared from freshly roasted coffee beans. During storage, the negative properties increased significantly (*P* < 0.05), but in Robusta beverages more than twice as much than in Arabica. In samples of both coffee varieties, however more intense in Robusta than in Arabica the rancid odor and flavor as well as staleness were already noticed after 9 months and increased significantly (*P* < 0.05) after 18 months of storage (Table 3).

## Discussion

Aromatic components are very important in coffee beverages, because they are the principal constituents of sensory experience for coffee consumers. Chemical transformation induced by storing roasted coffee beans are responsible for chemical and physical changes that may have a great influence on sensory quality of coffee brews (Toci et al. 2013). For instance, storage changes in composition of lipids may contribute to a loss of sensory quality of coffee beverages. Lipids can be hydrolyzed chemically or enzymatically and the rate at which these reactions occur depends on environmental and technological aspects, as well as availability of oxygen, moisture, temperature, and packaging material (Manzocco and Lagazio 2009). Roasting thermally inactivates hydrolytic enzymes in coffee beans. Afterwards, moisture and temperature are the main factors, which implement hydrolysis in roasted coffees by affecting the rates of all types of reactions. In addition, the presence of oxygen leads to the oxidation of unsaturated free fatty acids (FFA), which is responsible for the formation of a huge number of volatile compounds, loss of positive attributes such as brew-like, and formation of staleness. Both atmosphere and temperature in association with storage time influenced chemical and sensory changes. The use of inert atmosphere and low temperatures contributed to a slower degradation of FFA during storage period (Toci et al. 2013). Sour taste, bitterness, and undesired flavor notes were recognized as the most important attributes indicating the development of the sensory quality of coffee beverages upon storage (Ross et al. 2006; Manzocco and Lagazio 2009). The sensory evaluation of coffee beverages from the beans evaluated in our study showed an increase in the intensity of sour taste in Arabica after 18 months of storage and was about 60% higher in comparison to coffee beverage prepared from freshly roasted beans. In Robusta coffee, the perceived sourness doubled at the end of the storage compared to the preparation from freshly roasted beans. The bitterness increased by around 70% in Arabica, while in Robusta by approximately 20%. However, the bitter taste intensity in Robusta coffee beverages was much higher even at the first determination of the brew without storage. Furthermore, the decline of brew-like in odor and flavor in samples of both investigated coffee cultivars was between 40% and 60% in Arabica and Robusta, respectively. The staleness imperceptible in the coffee beverage brewed from freshly roasted beans was considerably perceived in both coffee drinks after storage.

Inert atmosphere, vacuum packaging, and also low storage temperatures are used and recommended by coffee industry to reduce degradation processes in coffees and give a rise in shelf life (Ross et al. 2006; Makri et al. 2011; Toci et al. 2013). But these procedures do not prevent chemical processes during storage; they only delay the consequences (Toci et al. 2013). Results of our study demonstrated that 9-month storage period of vacuum packed coffee beans are enough to make a great influence on sensory coffee cup quality. As time passes by, desirable positive attributes decreased and the negative ones were more intensely perceived in both coffee species. Therefore, more restrictive regulations for declaration and limitation of the best-before date and the roasting date of coffee are required.

## Conclusion

The conducted sensory evaluation demonstrated adverse effects on the quality of coffee beverages straight after 9 months storage of roasted coffee beans. After 18 months, the intensity of attributes which indicate oxidation processes increased. Consequently, we can conclude that sensory changes are likely during storage of roasted and vacuum-packed coffee beans. Therefore, we suggest testing the duration of storage of roasted coffee beans by the producers and recommend declaring the best-before date on the package.
